# Prophylaxis

**Published:** 1891-04

**Authors:** 


### Prophylaxis.

As a means of markedly invigorating the entire organism, and rendering thereby the system less prone to the influence of all external disease-producing agencies, an English physician reports, in a recent number of the *British Medical Journal*, the benefits of a daily morning cold bath. The bath used is *cold.* Often the ice has to be broken before the bath can be taken. Then the skin is toweled thoroughly. This physician says he never has a "cold;" never wears an overcoat—whatever the weather may be; has had no illness for many years; and would as soon think of stopping his meals, as to drop off the customary cold "tubbing."

In this connection might be mentioned the still more radical system of FATHER KNEIPP, the village priest of Voerishofen, Bavaria. It is said that he is well-regarded by the medical profession, and that many doctors go to him to learn his method of cure and care. The features of this so-called method are not at all new of themselves, and it is more the combination of requirements, together with the rigidness of procedure, which commands attention.

FATHER KNEIPP is simply a health reformer, and as such he declares strongly against all woolen garments next the skin. The underclothing he recommends is made of strong, coarse linen. Next of his requirements is the cold bath, which is to be taken quickly, and without subsequent drying of the skin. The linen garments are to be put on at once, while the moisture of the bath is still upon the body. Then comes the practice of walking or running barefooted in wet grass in cold weather, or in freshly-fallen snow. The village of Voerishofen lies in a valley where green meadows abound, and is therefore particularly adapted to to this form of exercise. After running in the wet in this fashion the patient puts on coarse linen socks, and boots, and walks briskly for a spell. Then as to internal attention, tea and coffee are absolutely prohibited, and animal food is discouraged. Bread, fruit, vegetables and milk are the substances permitted. "There are few better meals," says this man, " than plenty of fresh fruit and a piece of bread."

The patients are advised to drink before eating, never while eating, and after eating only when it seems necessary; and then moderately. Hard beds; cool, well-ventilated rooms; and the *use* of good things—without their *abuse*—quite completes this hygienic plan.—*Jour. Am. Med. Ass'n.*

